# BRCA1 functions as a novel transcriptional cofactor in HIV-1 infection

**DOI:** 10.1186/s12985-015-0266-8

**Published:** 2015-03-06

**Authors:** Irene Guendel, Beatrix W Meltzer, Alan Baer, Seth M Dever, Kristoffer Valerie, Jia Guo, Yuntao Wu, Kylene Kehn-Hall

**Affiliations:** National Center for Biodefense & Infectious Diseases, School of Systems Biology, George Mason University, Biomedical Research Lab, 10650 Pyramid Place, MS 1J5, Manassas, VA 20110 USA; Department of Radiation Oncology, Virginia Commonwealth University, Richmond, VA 23298 USA; Department of Pharmacology and Toxicology, Virginia Commonwealth University, Richmond, VA 23298 USA

**Keywords:** Antiviral, Small molecule inhibitor, Viral transcription, BRCA1, HIV-1, ATM, Phosphorylation, Post-translational modification

## Abstract

**Background:**

Viruses have naturally evolved elegant strategies to manipulate the host’s cellular machinery, including ways to hijack cellular DNA repair proteins to aid in their own replication. Retroviruses induce DNA damage through integration of their genome into host DNA. DNA damage signaling proteins including ATR, ATM and BRCA1 contribute to multiple steps in the HIV-1 life cycle, including integration and Vpr-induced G_2_/M arrest. However, there have been no studies to date regarding the role of BRCA1 in HIV-1 transcription.

**Methods:**

Here we performed various transcriptional analyses to assess the role of BRCA1 in HIV-1 transcription by overexpression, selective depletion, and treatment with small molecule inhibitors. We examined association of Tat and BRCA1 through *in vitro* binding assays, as well as BRCA1-LTR association by chromatin immunoprecipitation.

**Results:**

BRCA1 was found to be important for viral transcription as cells that lack BRCA1 displayed severely reduced HIV-1 Tat-dependent transcription, and gain or loss-of-function studies resulted in enhanced or decreased transcription. Moreover, Tat was detected in complex with BRCA1 aa504-802. Small molecule inhibition of BRCA1 phosphorylation effector kinases, ATR and ATM, decreased Tat-dependent transcription, whereas a Chk2 inhibitor showed no effect. Furthermore, BRCA1 was found at the viral promoter and treatment with curcumin and ATM inhibitors decreased BRCA1 LTR occupancy. Importantly, these findings were validated in a highly relevant model of HIV infection and are indicative of BRCA1 phosphorylation affecting Tat-dependent transcription.

**Conclusions:**

BRCA1 presence at the HIV-1 promoter highlights a novel function of the multifaceted protein in HIV-1 infection. The BRCA1 pathway or enzymes that phosphorylate BRCA1 could potentially be used as complementary host-based treatment for combined antiretroviral therapy, as there are multiple potent ATM inhibitors in development as chemotherapeutics.

## Background

Human immunodeficiency virus type 1 (HIV-1) is the etiological agent of the acquired immunodeficiency syndrome (AIDS). Currently approved combined antiretroviral therapy (cART) rely primarily on viral-based inhibitors, and present research efforts focus on finding new non-essential host targets that can provide viral inhibition without creating drug-resistance selective pressure on the virus. Therefore understanding the involvement of host factors might present a way to design better approaches to complement treatment and expand therapy considerations for multiple co-infections or HIV-associated malignancies.

The HIV-1 transactivator of transcription, Tat, is an essential regulatory protein that modulates the viral chromatin landscape and transcriptional activation by association with the transactivation response RNA loop region, TAR, present on the proviral 5′ region at the transcriptional initiation site (nt +1 to +57) on the HIV-1 long terminal repeat (LTR). The Tat/TAR complex is able to recruit various critical host factors including basal transcription factors TBP, TFIIB, TFIID, TFIIH, TAF55, and Sp1 [1]. Of importance, Tat/TAR recruits the pTEF-b complex (Cdk9/Cyclin T1) to the RNA polymerase II holoenzyme (RNAP II) that occupies the LTR [2-7]. pTEF-b is a ubiquitous positive acting elongation factor that actively phosphorylates the carboxyl-terminal domain (CTD) of RNAP II, and has been shown to be a critical cofactor for Tat activation of elongation [8-10].

Retroviruses induce DNA damage through integration of their genome into the host DNA. DNA damage signaling proteins including ATR, ATM and BRCA1, contribute to multiple steps in the HIV-1 life cycle, including integration and Vpr-induced G2/M arrest [11-14]. However, there have been no studies to date regarding the role of BRCA1 in HIV-1 transcription. Largely characterized in cancer, the breast cancer susceptibility gene, BRCA1, is a tumor suppressor protein that has implications in processes such as cell cycle, DNA repair, and transcription. These functions are accomplished by BRCA1 interacting with cellular transcription and host factors, in addition to stern regulatory mechanisms [15,16]. BRCA1 was first implicated in transcription when its C-terminus [amino acid (aa) 1560–1863] fused to Gal4 was able to activate transcription [17], with aa1760-1863 being the minimal transactivation domain (TAD). Within this TAD are two BRCA1 C-terminus (BRCT) motifs that are found in a large family of proteins important for DNA damage response, such as DNA ligase IV, p53BP1, and base excision response scaffold protein XRCC1 [18]. Since then, numerous other findings have served to strengthen the connection between transcription and BRCA1. For example, BRCA1 is part of the RNAP II holoenzyme complex [19-21]. BRCA1 also interacts with multiple cofactors and transcription factors including CBP/p300, Sp1, STAT1, estrogen receptor and BRG1 [22,23]. Among the genes found to be transactivated by BRCA1 are Mdm2, Bax, p21/Waf1, p27/Kip1, and GADD45α [24-30]. Moreover, BRCA1 has been shown to be in complex with pTEF-b, and Cyclin T1 has been shown to be an essential factor for BRCA1-dependent activation of RNAP II transcription [31]. Additionally, BRCA1 was reported to interact with the SWI/SNF catalytic core unit BRG1 [32]. In recent years, it has been further linked to chromatin alterations in cancer, including recruitment to sites of DNA repair, indicating a direct function of BRCA1 in transcriptional control through chromatin structure modulation [33-36].

Taking into account these findings and its well-documented involvement in transcriptional regulation and chromatin remodeling capabilities, we were interested in determining whether BRCA1 participates in HIV-1 Tat-dependent transcription. Here, we have demonstrated BRCA1 enhancement of HIV-1 Tat-dependent transcription through gain-of-function and loss-of-function studies. Tat was detected in complex with BRCA1 at aa504-802 through *in vitro* binding assays and co-immunoprecipitated with BRCA1. Additionally, inhibition of the effector kinase ATM, but not Chk2, resulted in transcriptional decrease by loss of BRCA1 from the viral promoter. Therefore, targeting the host BRCA1 activation pathway can serve as an attractive strategy for the development of novel host-based therapeutics that target HIV-1 viral transcription.

## Results and discussion

### BRCA1 enhances HIV-1 Tat-dependent transcription

The molecular function of BRCA1 has been the subject of intensive studies since it was cloned in 1994 [37]. Primarily in cancer, it has been characterized as a multifaceted tumor suppressor protein due to its role in cell cycle progression, DNA repair and DNA damage response processes, transcription, RNAi pathway regulation, and apoptosis [23,38]. Limited studies have linked BRCA1 to HIV-1. Initially, Zimmerman *et al*. [13] showed BRCA1-H2AX foci formation was required for Vpr-induced G_2_ arrest. Later, this same group further elaborated on the role of BRCA1 in infection by showing Vpr-induced ATR-dependent activation of BRCA1 at serine substrate S1423 as a response to genotoxic stress, suggesting a model for Vpr-induced apoptosis via transcriptional regulation of the BRCA1 target gene, GADD45α [11]. Around the same time, Coberley *et al*. [12] demonstrated alterations of various genes contributing to cell cycle transition at the G_2_/M checkpoint through a temporal genetic network study in mock or R5-tropic HIV-1 infected primary macrophages. Specifically, infection activated mediators of cell cycling at different times during infection including BRCA1 (intermediate), and GADD45α (late). Furthermore, BRCA1 may function as a transcriptional coactivator or corepressor, a function that varies depending on its ability to recruit both the basal transcription machinery and proteins implicated in chromatin remodeling [32,39]. Consequently, we were interested in characterizing BRCA1 function in HIV-1 transcription.

**Figure 1:**
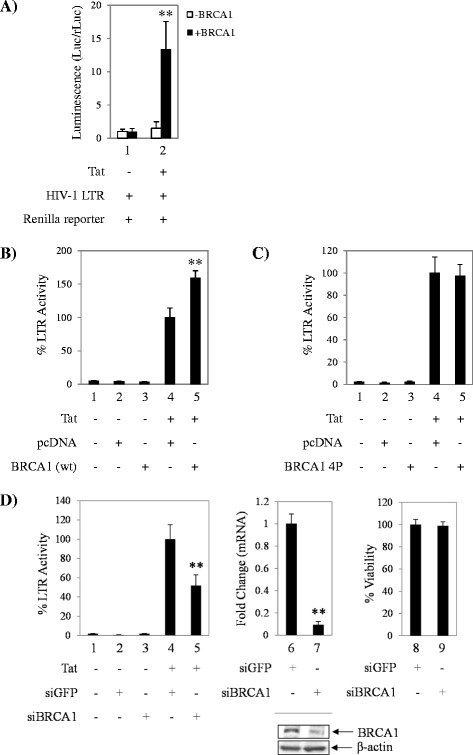
**HIV-1 Tat-dependent transcription is more efficient in cells containing BRCA1. A**. UWB1.289 BRCA1 null and UWB1.289 + BRCA1 cells and were co-transfected with pcDNA or pcTat, LTR-Luc, and CMV-Luc (Renilla) plasmid DNA. Dual-Glo luciferase assay was performed 48 hours later as described by the manufacturer. Raw data was normalized to Renilla luciferase expression in both cell lines, fold changes were calculated against pcDNA (−Tat) condition for each cell line. **B**. TZM-bl cells were co-transfected with pcDNA or pcTat, and BRCA1 (wild-type) plasmid DNA. Bright-Glo luciferase assay was performed 48 hours pos-transfection as described by the manufacturer. Cells containing pcTat and pcDNA were used as baseline value for Tat-dependent LTR activation. **C**. TZM-bl cells were co-transfected with pcDNA or pcTat, and BRCA1 4P (mutant) plasmid DNA (S1387A, S1423A, S1457A, and S1524A). Bright-Glo luciferase assay was performed 48 hours post-transfection. Cells containing pcTat and pcDNA were used as baseline value for Tat-dependent LTR activation. **D**. TZM-bl cells were co-transfected with pcTat or siRNA against GFP (control) and BRCA1. Bright-Glo luciferase assay was performed 48 hours post-transfection (left panel). Cells containing pcTat and siGFP were used as baseline value for Tat-dependent LTR activation. BRCA1 depletion was confirmed by qRT-PCR and western blot (middle panel and inset). Fold changes against siGFP were calculated relative to Actin using the ΔΔCt method. CellTiter-Glo cell viability assay (right panel) was performed as described by the manufacturer and viability normalized to cells transfected with siGFP. Transfection assays were performed in triplicate and data plotted represents averaged data of two independent experiments. Error bars show the standard error of two averaged independent measurements. Viability assays were performed in triplicate. Western blots were performed for two independent experiments. *Double asterisk* indicates statistically significant difference *p* ≤ 0.01.

First we started our functional transcription studies in cells that have a null background for BRCA1 expression. UWB1.289 cells are derived from ovarian cancer in a germ line BRCA1 mutation carrier and lack expression of BRCA1, while UWB1.289 + BRCA1 cells are a stable UWB1.289 derivative cell line carrying a pcDNA3 plasmid coding for wild-type BRCA1 [40]. Here we co-transfected both set of cells with HIV-1 LTR-Luc, pcDNA or Tat, and Renilla reporter plasmid as a control for transfection efficiency between cell lines. Based on scored luciferase activity, results in Figure 1A indicate that there is ~13-fold difference in Tat transactivation of the HIV-1 LTR in cells expressing BRCA1 (lane 2, black bar) when compared to the BRCA1 null cells (lane 2, white bar). Next, we were interested in assessing the effect of BRCA1 overexpression in the context of integrated proviral DNA. To this end, we co-transfected pcDNA or Tat, and BRCA1 into TZM-bl cells followed by measurement of the HIV-1 LTR activity through luciferase assays. The TZM-bl cell line is a widely used reporter system for the study of Tat-dependent transcription as it harbors the luciferase gene under the control of an integrated HIV-1 5′ LTR. Results in Figure 1B show a significant transcriptional enhancement (~60% increase) when BRCA1 is overexpressed in comparison to pcDNA-transfected cells (compare lanes 5 and 4). Importantly, we did not observe any effects of BRCA1 overexpression in the absence of Tat, suggesting a Tat-dependent effect (lanes 2 and 3). Given that BRCA1 is activated by phosphorylation [23], which has also been noted to occur during HIV-1 infection [11], we assessed the importance of BRCA1 phosphorylation in the enhancement of HIV-1 transcription. To this end, we utilized a BRCA1 mutant construct with amino acid substitutions for the ATR/ATM target serine residues S1387A, S1423A, S1457A, and S1524A (BRCA1 4P). Similarly, TZM-bl cells were co-transfected with Tat or pcDNA, and BRCA1 4P for 48 hours, with subsequent Tat-dependent HIV-1 LTR-driven luciferase expression quantification. Results in Figure 1C showed no transcriptional enhancement between cells containing control DNA or BRCA1 4P (lanes 4 and 5), indicating that BRCA1 activation by phosphorylation is important for the enhancement of Tat dependent LTR transcription. Likewise, we did not see any effects of BRCA1 4P overexpression in our basal controls (lanes 2 and 3).

Based on these findings, we next wanted to examine the functional consequences of BRCA1 selective depletion in Tat-dependent transcription. To assess the effect of BRCA1 knockdown in HIV-1 transcription, we first screened various siRNAs against BRCA1 and selected the one producing more effective depletion results (data not shown). TZM-bl cells were co-transfected with Tat and siRNA against BRCA1 or GFP as a non-specific control, and luciferase assays were performed 48 hours post-transfection. Results in Figure 1D show that luciferase activity was significantly reduced by ~49% in cells transfected with siBRCA1 compared to cells transfected with siGFP (compare lanes 4 and 5). As additional controls, BRCA1 levels in these cells were assayed by qRT-PCR and western blot. BRCA1 was successfully repressed (~90%) at the transcriptional level (compare lanes 6 and 7) and protein levels were decreased by ~68% (inset western blot panel). To discard the possibility that the decrease in Tat-dependent transcription was due to cytotoxic effects upon BRCA1 knockdown, we performed viability assays on these samples. No changes in cell viability were observed between samples transfected with control siRNA or BRCA1 siRNA (compare lanes 8 and 9), indicating that the transcriptional efficiency loss is specific to BRCA1 selective depletion. Collectively, these data support the permissive role of BRCA1 for Tat-dependent HIV-1 transcription.

### BRCA1 is in complex with Tat

**Figure 2:**
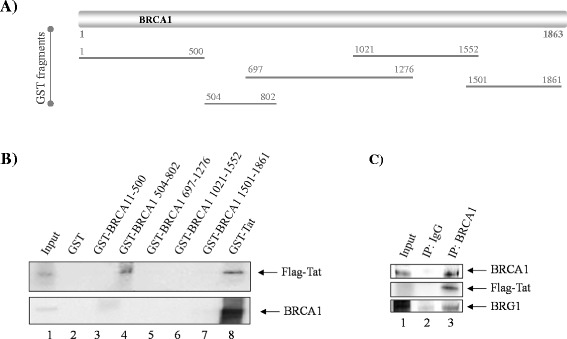
**BRCA1 associates with Tat. A**. Schematic of five GST-BRCA1 fragments spanning the whole length protein. **B**. TZM-bl cells were transfected with Flag-Tat plasmid DNA for 48 hours. One milligram of whole cell protein extract was incubated with 1 μg GST-BRCA1 constructs overnight and processed as described in the Methods section. Samples were analyzed by western blot (top panel WB: anti-Flag, bottom panel WB: anti-BRCA1). **C**. TZM-bl cells were transfected with Flag-Tat plasmid DNA for 48 hours. One milligram of TZM-bl whole cell protein extract was immunoprecipitated with anti-BRCA1 and anti-IgG antibodies and analyzed by western blot (top panel WB: anti-BRCA1, middle panel WB: anti-Flag, bottom panel WB: anti-BRG1). Western blots are representative of two independent experiments.

Collectively, molecular studies have shown ~1,500 interactions between HIV-1 and human host proteins [41]. In terms of Tat, the most studied interaction is its association with the pTEF-b Cyclin T1 subunit by which it exerts Tat-dependent transcriptional activation of the HIV-1 promoter [1]. Because of the fact that Tat is known to be in complex with various transcription factors and host transcriptional machinery, we were interested in determining whether these proteins associate. To this end, GST-BRCA1 constructs spanning the full length of the protein (Figure 2A) and GST-Tat were utilized in a pull-down assay from Flag-Tat transfected TZM-bl whole cell lysates. TZM-bl cells were chosen as they express robust levels of BRCA1 (see Figure 1D), and are our base cell line for our transcriptional assays. Beads bound to GST protein (lane 2, panel B) were used as a background control. Membranes were probed with antibodies against Flag (top panel) and BRCA1 (bottom panel). Western blot results in Figure 2B indicate that the only detectable association between the GST-BRCA1 fragments and Flag-Tat occurs at the aa504-802 region (lane 4, top panel), while GST-Tat inversely confirmed the BRCA1-Tat association observed with full length endogenous BRCA1 present in the TZM-bl lysate (lane 8, bottom panel). In addition, we saw GST-Tat binding to the Flag-Tat present in the cell lysate (lane 8, top panel), doubling as a positive binding control given that Tat exists as a Zn^2+^- or Cd^2+^-linked dimer [42]. To further corroborate this interaction, whole cell extracts from Tat-transfected TZM-bl cells were immunoprecipitated with BRCA1 or IgG and western blotted with anti-BRCA1, Flag, and BRG1 antibodies. Results in Figure 2C show that BRCA1-Tat interaction was observed specifically with the BRCA1 immunoprecipitation and not with the IgG. Probing against BRG1 was used as an immunoprecipitation control since it has been shown to be a BRCA1-binding partner [32]. Collectively, these results indicate that physical interaction of BRCA1-Tat is detectable at the aa504-802 region of BRCA1, suggesting that these proteins are associated and accordingly, supporting BRCA1 participation in Tat-dependent transcription.

### BRCA1 status affects enhancement of Tat-dependent transcription during infection with pseudotyped particles

**Figure 3:**
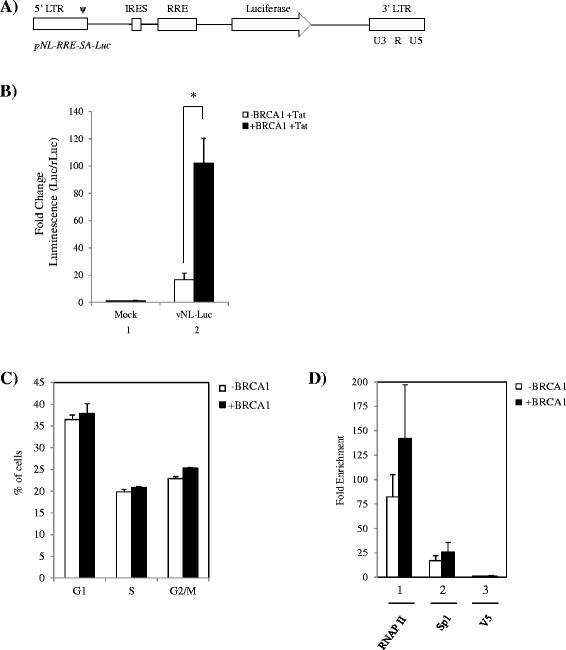
**BRCA1 status affects enhancement of Tat-dependent transcription during infection with pseudotyped particles. A**. Schematic depicting the LTR-driven reporter plasmid *pNL-RRE-SA-Luc*. **B**. UWB1.289 BRCA1 null and UWB1.289 + BRCA1 cells and were co-transfected with pcTat and CMV-Luc (Renilla) plasmid DNA 24 hours prior to infection with LTR-driven reporter viral particles. Dual-Glo luciferase assay was performed 24 hours post-infection as described by the manufacturer. Raw data was normalized to Renilla luciferase expression in both cell lines, fold changes were calculated against + Tat mock infected cells (lane 1). Transfection and infection assays were performed in triplicate and data plotted represents averaged data of two independent experiments. *Single asterisk* indicates *p* < 0.05. **C**. UWB1.289 BRCA1 null and UWB1.289 + BRCA1 cells were infected with VSVG-pseudotyped NL4-3. Cells were collected 48 hours post-infection, stained with propidium iodine, and analyzed by flow cytometry. Results are representative of three biological replicates. **D**. Cells were infected as described above and collected at 48 hours post-infection for ChIP analysis. Antibodies used for ChIP were anti-RNA polymerase II (RNAP II, 10 μg), anti-Sp1 (10 μg), and anti-V5 (10 μg). Quantitative PCR was performed using SYBR Green PCR Master Mix to analyze immunoprecipitated material.

To further discern the role of BRCA1 in Tat-dependent transcription, we generated pseudotyped HIV-1 LTR-driven reporter viral particles. Figure 3A shows the general structure of the LTR-driven reporter plasmid *pNL-RRE-SA-Luc* that was used to incorporate the luciferase reporter gene. To assay the impact of BRCA1 status in Tat-dependent transcription in a live infection, we utilized the UWB1.289 and UWB1.289 + BRCA1 cells for their unique ability to provide clean contrasts of BRCA1 expression. Cells were co-transfected with pcTat and Renilla reporter 24 hours prior to infection. Next, cells were infected and processed for luciferase assays 24 hours post-infection to allow for sufficient proviral integration and transcription to take place. Results in Figure 3B indicate that in vNL-Luc infected cells, LTR activation was observed at a lower level in –BRCA1 cells (lane 2, white bar) when compared to + BRCA1 cells (lane2, black bar), which exhibited a ~8-fold increase in LTR activity over its –BRCA1 counterpart. No significant background activation was detected in + Tat mock infected cells (lane 1). Collectively, these findings support our initial assays performed in these cells with a non-integrated HIV-LTR reporter plasmid (Figure 1A), where we detected a more tolerant environment for Tat-dependent transcription to occur in the presence of BRCA1. Moreover, these results provide further support that the enhancement of transcription by BRCA1 is Tat-dependent.

Given the importance of BRCA1 to the cell cycle and the fact that HIV-1 transcription has been shown to be enhanced in the G2 phase of the cell cycle [11-13,23,43,44], experiments were performed to determine if UWB1.289 cells had a difference in cell cycle distribution. It has been previously shown that UWB1.289 cells display no difference in cell cycle profile in the absence of stress, but when cells were subjected to irradiation, a G2/M arrest was observed with the BRCA1 complemented cells [40]. To determine if any alterations in cell cycle were occurring in the UWB1.289 and UWB1.289 + BRCA1 cells following HIV infection, cell cycle analysis using propidium iodine staining and flow cytometry was performed. No changes in cell cycle distribution between these two cell derivatives were observed (Figure 3C). As Sp1 is a well-known interacting partner of BRCA1, the binding of Sp1 to the LTR in the presence and absence of BRCA1 was assessed by chromatin immunoprecipitation. ChIP assays from vNL-Luc infected –BRCA1 and + BRCA1 cells were performed using antibodies against RNAP II (positive control), V5 (negative control) and Sp1. There was no significant difference in Sp1 or RNAP II binding to the LTR between the two cell populations (Figure 3D), indicating that differential binding of Sp1 to the LTR is not a contributing factor to the decreased LTR activation observed in the –BRCA1 cells. Collectively, these results suggest that BRCA1 plays a direct and critical role in Tat-dependent HIV transcription independent of cell cycle effects.

### Small molecule inhibition of BRCA1 gene expression affects HIV-1 Tat-dependent transcription

Currently, there are no therapeutic agents specifically targeting BRCA1. However recently, it has been documented that curcumin reduces expression of the BRCA1 gene by histone acetylation impairment at the BRCA1 promoter [45]. Generally, curcumin is categorized as a multifactorial compound characterized by tolerable doses linked to antioxidant therapy, anti-tumor effects, and anti-viral effects [45-50]. Moreover, curcumin has been shown to suppress pathways concomitant to HIV-1 replication and HIV-1 associated neurocognitive disorders (HAND); and when administered as adjuvant therapy to existing cART, curcumin has been shown to enhance the protease inhibitor indinavir antiretroviral activity in persistently infected cells [51-54]. In addition, a recent study demonstrated multi-pathway involvement in curcumin-induced inhibition of Tat-dependent transcription [55]. Here, the authors showed modulation of the HDAC1/NF-κB pathway used by HIV-1 to exert chromatin remodeling at the viral promoter and further promoter activation by NF-κB. Despite curcumin not being BRCA1-specific, we were interested in using the compound as a model for small molecule inhibition of BRCA1.

**Figure 4:**
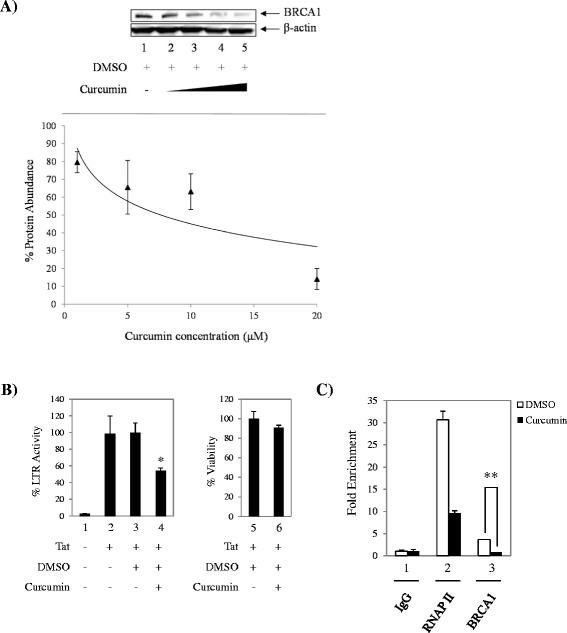
**Curcumin decreases BRCA1 occupancy at the HIV-1 LTR. A**. TZM-bl cells were transfected with pcTat and treated the next day with vehicle (DMSO) or a titration of curcumin (0.5, 1, 10, and 20 μM). Samples were analyzed by western blot. Inset depicts a dose–response curve of BRCA1 protein abundance *versus* curcumin treatment based on the average densitometry counts with error bars representing standard error of three independent measurements. Western blot is representative of three independent experiments. Densitometry counts were taken from three independent treatments to acquire a dose–response curve of BRCA1 expression inhibition (inset plot) **B**. TZM-bl cells were transfected with pcTat and treated the next day with DMSO or 20 μM curcumin. Bright-Glo luciferase assays and CellTiter-Glo cell viability assays were performed 24 hours post-treatment as described by the manufacturer. Data was normalized to cells containing Tat and treated with DMSO as baseline for Tat-dependent LTR activation. Transfection and treatment assays were performed in triplicate and data plotted represents averaged data of two independent experiments. Error bars show the standard error of two averaged independent measurements. Viability assays were performed in triplicate. **C**. TZM-bl cells were transfected with pcTat and treated the next day with DMSO or curcumin (20 μM) for 24 hours prior to being collected for ChIP analysis. Antibodies used for ChIP were anti-BRCA1 (10 μg), anti-IgG (10 μg), and anti-RNA polymerase II (RNAP II, 10 μg). Quantitative PCR was performed using SYBR Green PCR Master Mix to analyze immunoprecipitated material. *Single asterisk* indicates *p* < 0.05 and *double asterisk* indicates statistically significant difference *p* ≤ 0.01.

To confirm previously published findings in our cell-based system, TZM-bl cells were treated for 24 hours with vehicle (DMSO) or a titration of curcumin determined from concentrations used in the literature (0.5, 1, 10, and 20 μM). Western blot was then performed using anti-BRCA1 and anti-β-actin antibodies to determine the expression levels of BRCA1. Results in Figure 4A show a dose-dependent loss of BRCA1 expression with curcumin treatment when compared to DMSO (compare lanes 1 to 2–5). Next, we confirmed Tat-dependent transcription inhibition by treating Tat-transfected TZM-bl cells with 20 μM of curcumin. Results in Figure 4B show a significant decrease in transcription (~46%) with curcumin when compared to DMSO (compare lanes 3 and 4) that is specific to treatment and not due to compound cytotoxicity (compare lanes 5 and 6) and likens previously published data [55]. We next sought to determine if BRCA1 was present at the HIV-1 LTR, and if curcumin treatment could modulate its binding. ChIP assays from Tat-transfected DMSO- or curcumin-treated (20 μM) TZM-bl cells were performed using antibodies against RNAP II (positive control), IgG (negative control) and BRCA1. Interestingly, we observed the presence of BRCA1 at the viral promoter (Figure 4C). To our best knowledge, this is the first instance that BRCA1 has been detected at the HIV-1 LTR. Conversely curcumin treatment resulted in a significant ~2.9-fold loss of BRCA1 occupancy from the activated HIV-1 LTR when compared to the DMSO control (lane 3). Collectively, these results are suggestive of BRCA1 playing a role in HIV-1 Tat dependent transcription, and implies the use of chemotherapeutic agents treating BRCA1 as a druggable target.

### Inhibition of upstream BRCA1 phosphorylation effectors, ATR/ATM, decreases Tat-dependent transcription

**Figure 5:**
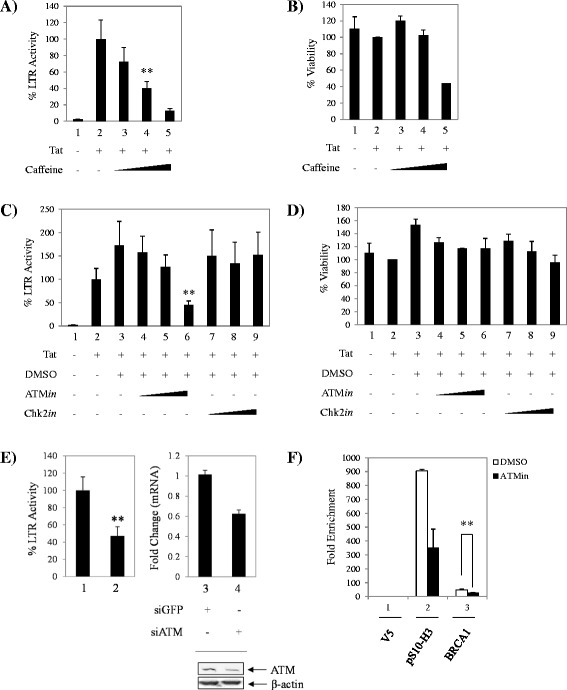
**Inhibition of upstream BRCA1 phosphorylation effectors ATR/ATM, decreases Tat-dependent transcription. A**. TZM-bl cells were transfected with pcTat and treated the next day with vehicle (water) and a titration of caffeine (500 μM, 2 mM and 5 mM). Bright-Glo luciferase assays were performed 48 hours post-treatment. Data was normalized to cells containing Tat and treated with DMSO as baseline for Tat-dependent LTR activation. **B**. CellTiter-Glo cell viability assays were performed 48 hours post-treatment. Data was normalized as in panel A. **C**. TZM-bl cells were transfected with pcTat and treated the next day with vehicle (DMSO) and a titration of ATM or Chk2 inhibitors (ATM*in* and Chk2*in*) at 0.1 μM, 1 μM and 10 μM. Bright-Glo luciferase assays were performed 48 hours post-treatment. Data was normalized as in panel A. **D**. CellTiter-Glo cell viability assays were performed 48 hours post-treatment. Data was normalized as in panel A. **E**. TZM-bl cells were co-transfected with pcTat or siRNA against GFP (control) and ATM. Bright-Glo luciferase assays were performed 48 hours post-treatment. Cells containing pcTat and siGFP were used as baseline value for Tat-dependent LTR activation. ATM depletion was confirmed by qRT-PCR and western blot (right panel and inset). Fold changes against siGFP were calculated relative to Actin using the ΔΔCt method. **F**. TZM-bl cells were transfected with pcTat and treated the next day with DMSO or ATM inhibitor (10 μM) for 48 hours prior to being collected for ChIP analysis. Antibodies used for ChIP were anti-BRCA1 (10 μg), anti-V5 (10 μg), and anti- pS10-H3 (10 μg). Transfection and treatment assays were performed in triplicate and data represents averaged data of two independent experiments. Viability assays were performed in triplicate. Error bars show the standard error of two averaged independent measurements. *Double asterisk* indicates statistically significant difference *p* ≤ 0.01.

BRCA1 contains a serine cluster domain (SCD) spanning aa1280-1524, a common motif present in ATR/ATM protein targets [56]. ATR phosphorylates BRCA1 on serine 1423 in response to UV damage or HU-induced replication arrest [57], while ATM-mediated phosphorylation events at serines 1387, 1423, 1457 and 1524, have been characterized in response to ionizing radiation-induced damage [58,59]. While the Chk2 phosphorylation site is not located within the SCD region, its phosphorylation of serine 988 occurs in response to the same stressors as with ATM [60]. Because cellular stress was shown to be highly conducive to HIV-1 replication [61], we next looked at the effect of ATR/ATM inhibition on Tat-dependent HIV-1 transcription. TZM-bl cells were transfected with Tat and treated the next day with vehicle or a titration of caffeine. Caffeine is a methylxanthine that has been used extensively to study ATR/ATM signaling as a natural inhibitor of these kinases [62]. Forty eight hours post-treatment the cells were subjected to luciferase assays. As can be observed in Figure 5A, there is a dose-dependent decrease in HIV-1 transcription with increasing concentrations of caffeine of up to ~88% inhibition when compared to vehicle (compare lane 2 to 3–5). To confirm compound inhibition specificity, we performed cell viability assays of treated cells to measure inhibitor-induced cell death. Results in Figure 5B show that caffeine was not toxic at 500 μM or 2 mM. Working concentrations in published literature use caffeine up to 10 mM, however we observed that in our system, 5 mM caffeine decreased cell viability by ~60% (lane 5). Thus, treatment with 2 mM caffeine results in a viable ~60% Tat-dependent transcriptional activity decrease without exhibiting cytotoxicity.

Given that caffeine is a generic inhibitor, we wanted to explore treatment with a more specific compound. To confirm the importance of ATM in this process, the small molecule ATM inhibitor, KU55933 (which we will refer to henceforth as ATM*in*) was utilized. This inhibitor has been shown to specifically inhibit ATM in the low IC_50_ of 12.9 nM without inhibiting ATR at doses of up to 100 μM [63]. As Chk2 also phosphorylates BRCA1 in response to DNA damage [64], a Chk2 inhibitor (Chk2*in*) was also tested. TZM-bl cells were transfected with Tat and treated the next day with vehicle or a titration of ATM*in* or Chk2*in*. Forty eight hours post-treatment the cells were subjected to both luciferase (Figure 5C) and viability assays (Figure 5D). The results showed a dose-dependent Tat-dependent LTR transcriptional inhibition of up to ~55% in the presence of ATM*in* but not Chk2*in* (compare lanes 6 and 9, panel C), demonstrating ATM kinase specificity in this inhibitory process. Both inhibitors showed no cytotoxicity. To further confirm the specific participation of ATM in Tat-dependent transcription, we performed ATM selective depletion in Tat-transfected TZM-bl cells. Results in Figure 5E indicate that upon ATM knockdown, Tat transactivation of the viral promoter is decreased by ~45%. As additional controls, ATM levels in these cells were assayed by qRT-PCR and western blot. ATM expression was successfully decreased (~40%) at the transcriptional level (compare lanes 3 and 4) and protein levels were decreased by ~54% (inset panel) as confirmed by western blot using antibodies against ATM and β-actin as a loading control.

In order to functionally link the inhibitor study findings to BRCA1, ChIP assays from Tat-transfected DMSO- and ATM*in*-treated TZM-bl cells were performed using antibodies against Histone H3 phosphorylated at serine 10 [pS10-H3 (positive control)], V5 (negative control), and BRCA1. Results in Figure 5F reveal total BRCA1 (lane 3, compare white and black bars) occupancy loss (~2-fold) from the activated HIV-1 LTR with ATM*in* treatment when compared to the DMSO control. It is important to note that we also observed a decrease in pS10-H3 binding to the LTR following ATM*in* treatment as well as a decrease in RNAP II binding following curcumin treatment (Figure 4C). These results indicate that these treatments are affecting more than just BRCA1 binding to the LTR. As we hypothesize that BRCA1 may be acting as a scaffold protein, there are likely multiple LTR binding factors that will be influenced if BRCA1 leaves the LTR. However, we cannot rule out a global change at the promoter after treatments that is affecting multiple binding partners. Taken together, these results suggest that BRCA1 phosphorylation plays a role in Tat-dependent transcription as observed indirectly by the inhibition of upstream BRCA1 activators. Also, they are suggestive of ATM-dependent BRCA1 phosphorylation requirement for its recruitment to the HIV-1 LTR in the presence of Tat. Moreover, these results show that LTR transcription is potently and specifically inhibited by ATM*in*. These observations are correlated with various studies implicating these kinases in HIV-1 infection [14,65-68]. For example, caffeine and caffeine-related methylxanthines, including FDA-approved theophylline, have been used to inhibit HIV-1 integration in primary cells [65]. Similarly, HIV-1 IN has been shown to stimulate an ATM-dependent DNA damage response and that in the absence of this enzyme, cells are sensitized to retroviral-induced death [67]. Thus, treatment with ATM*in* suppressed viral replication, not only of wild-type, but drug-resistant HIV-1. Of interest for the present study, caffeine has been found to prevent pTEF-b dissociation from its 7SK snRNP inactive complex, illustrating an additional mechanism for its inhibition of Tat-dependent transcription [69].

### BRCA1 is present at the HIV-1 LTR in HIV infected T-cells

**Figure 6:**
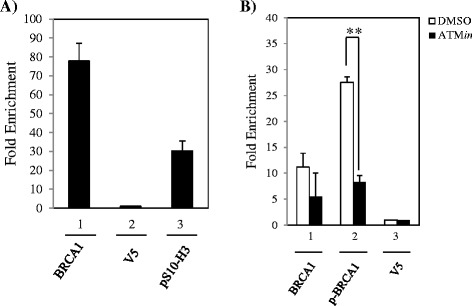
**BRCA1 is present at the HIV-1 LTR in HIV infected T-cells. A**. CEM cells were infected with NL4-3 virus (p24 = 5000 pg/ml) for 4 hours and collected 72 hours post-infection for ChIP analysis. Antibodies used for ChIP were anti-BRCA1 (10 μg), anti-V5 (10 μg), and anti-histone H3-phosphorylated at S10 (pS10-H3, 5 μg). Quantitative PCR was performed using SYBR Green PCR Master Mix to analyze immunoprecipitated material. **B**. CEM cells were pre-treated with DMSO or 10 μM ATM inhibitor (ATM*in*) for 2 hours. Cells were then infected with NL4-3 virus (p24 = 5000 pg/ml) for 4 hours, followed by post-treating the cells with DMSO or ATM*in*. Cells were collected 72 hours post-infection for ChIP analysis. Antibodies used for ChIP were anti-BRCA1 (10 μg), p-BRCA1 S1423 (10 μg), and anti-V5 (10 μg). Quantitative PCR was performed using SYBR Green PCR Master Mix to analyze immunoprecipitated material. *Double asterisk* indicates statistically significant difference *p* ≤ 0.01.

Finally we aimed to confirm our findings in a T-cell model of infection. To this end, CEM T-cells were infected with HIV and BRCA1 occupancy of the LTR assessed by chromatin immunoprecipitation. ChIP assays from HIV (NL4-3) infected CEM T-cells were performed using antibodies against pS10-H3 (positive control), V5 (negative control), and BRCA1. Indeed, we observed BRCA1 present at the LTR in HIV infected T-cells (Figure 6A). Next we examined if inhibition of BRCA1 phosphorylation through the use of the ATM*in* would alter BRCA1 binding to the LTR. CEM cells were pre-treated with ATM*in* for 2 hours prior to infection. ATM*in* was also added following infection. Interestingly, no significant difference in BRCA1 binding to the LTR was observed after ATM*in* treatment (Figure 6B). However, a decrease in p-BRCA1 (S1423) was observed after ATM*in* treatment, indicating that there was a shift in the form of BRCA1 bound at the LTR following ATM*in* treatment. Collectively these results indicate that BRCA1 is present at the HIV-1 LTR in a highly relevant model of HIV infection.

## Conclusions

Our data has shown that BRCA1 functions as an enhancer of HIV-1 transcription through gain-of-function and loss-of-function studies. We have shown that BRCA1 region aa504-802 associates with Tat, encouraging the idea of its participation in Tat-dependent transcription. We have also used small molecule inhibitors as tools to assess the inhibitory effect of its targets on Tat-dependent HIV-1 5′ LTR transcription, highlighting the viability of therapeutic approaches targeting host cell proteins. BRCA1 phosphorylation in HIV-1 infected cells has primarily been described as a function of ATR as the effector kinase and as a DNA damage response event [11]. These data explored the effects of ATR/ATM-mediated phosphorylation of BRCA1 in Tat-dependent transcription. More importantly, we have shown for the first time that BRCA1 is present at the HIV-1 LTR in infection and that LTR occupancy by the phosphorylated species is decreased upon treatment with ATM*in*.

## Methods

### Cell culture and reagents

TZM-bl cells are engineered HeLa cells that express CD4, CCR_5_, and CXCR_4_ and contain integrated reporter genes for Firefly luciferase and β-galactosidase under the control of an HIV-1 long terminal repeat [70,71]. These cells were cultured to confluency in DMEM supplemented with 10% heat-inactivated FBS, 1% L-glutamine, and 1% streptomycin/penicillin (Gibco/BRL, Gaithersburg, MD, USA). UWB1.289 cells are derived from ovarian cancer in a germ line BRCA1 mutation carrier and lack expression of BRCA1 [40]. UWB1.289 + BRCA1 cells are a stable UWB1.289 derivative cell line carrying a pcDNA3 plasmid coding for wild-type hemagglutinin-tagged BRCA1 [72]. Cells were purchased from the American Type Culture Collection (Manassas, VA, USA) and were maintained in 1:1 RPMI 1640/MEGM (Lonza, Walkersville, MD, USA) supplemented with 3% fetal bovine serum. CEM is an uninfected T-cell line and is grown in RPMI media containing 10% FBS, 1% L-glutamine, and 1% streptomycin/penicillin.

### Protein extracts and immunoblotting

Cells were collected, washed once with PBS and pelleted. For immunoprecipitation, cells were lysed in a buffer containing Tris–HCl pH 7.5, 120 mM NaCl, 5 mM EDTA, 0.5% NP-40, 50 mM NaF, 0.2 mM Na3VO4, 1 mM DTT and one tablet complete protease inhibitor cocktail per 50 ml. Lysis was performed under ice-cold conditions, incubated on ice for 30 min and spun at 4°C for 5 min at 14,000 rpm. The protein concentration for each preparation was determined with by Bradford assay (Sigma Aldrich, St. Louis, MO, USA). For immunoblotting, lysis buffer consisted of a 1:1 mixture of T-PER reagent (Pierce, Rockford, IL, USA) and 2X Tris-glycine SDS sample buffer (Novex, Life Technologies, Carlsbad, CA, USA), 33 mM DTT, and protease and phosphatase inhibitor mixture (1X Halt mixture, Pierce). Cells were collected directly in lysis buffer and boiled for 10 min. Cell extracts were resolved by SDS-PAGE on a 4-20% tris-glycine gel (Invitrogen, Life Technologies). Proteins were transferred to PVDF membranes by overnight transfer as described by the manufacturer (Invitrogen, Life Technologies). Membranes were blocked with PBS 0.1% Tween-20 + 3% BSA. Primary antibodies against specified proteins were incubated with the membrane in blocking solution overnight at 4°C. Antibodies against BRCA1 (sc-642) and BRG1 (sc-10768) were purchased from Santa Cruz Biotechnology (Santa Cruz, CA, USA). The ATM (2873) antibody was purchased from Cell Signaling (Beverly, MA, USA). The β-actin antibody (ab49900) was purchased from Abcam (Boston, MA, USA). Anti-Flag (F3165) was purchased from Sigma Aldrich. Membranes were washed twice with PBS + 0.1% Tween-20 and incubated with HRP-conjugated secondary antibody for 1 hour in blocking solution. Presence of secondary antibody (#32430 and #32460, Pierce) was detected by SuperSignal West Dura Extended Duration Substrate (Pierce). Luminescence was visualized on a Molecular Imager ChemiDoc XRS system Bio-Rad station (Bio-Rad, Hercules, CA, USA).

### Small molecule compounds

The ATM kinase inhibitor (ATM*in*) *2-morpholin-4-yl-6-thianthren-1-yl-pyran-4-one* (KU55933) and Chk2 kinase inhibitor (Chk2*in*) 2*-(4-(4-chlorophenoxy)phenyl)-1H-benzimidazole-5-carboxamide*, were purchased from EMD4 Biosciences (Gibbstown, NJ, USA). Caffeine was purchased from Sigma Aldrich. Curcumin was purchased from Santa Cruz Biotechnology (sc-200509). All inhibitors were prepared in 10 mM stock solution dissolved in DMSO.

### Transfections

UWB1.289 and UWB1.289 + BRCA1 cells were seeded in a 96-well plate and co-transfected with 0.5 μg of *pRL-CMV-luciferase* reporter (Renilla), HIV-1 LTR-luciferase reporter (Firefly), and pcTat_101_. The Renilla reporter plasmid was co-transfected to allow correction for differences in transfection efficiency. For siRNA transfection, TZM-bl cells were co-transfected in a 96-well plate with pcTat_101_ (0.5 μg) and siRNA against GFP (#P-002048-01-20, Dharmacon, Lafayette, CO, USA), Hs_BRCA1_15 or Hs_ATM_5 (#SI02664368 and #SI00299299, Qiagen, Valencia, CA, USA), using DharmaFECT Duo (Dharmacon). For other transcriptional assays, TZM-bl cells were co-transfected in a 96-well plate with pcTat_101_, BRCA1 (wild-type) [73], or BRCA1 4P (S1387A/S1423A/S1457A/ S1524A) mutant [constructed by swapping a cDNA fragment harboring the S1387A and S1423A mutations into the wild-type plasmid followed by adding additional mutations with sequential rounds of QuikChange site-directed mutagenesis (Stratagene, Santa Clara, CA, USA) using primer sets: 5′-GCAGTATTAACTGC ACAGAAAAGTAGTG-3′ and 5′-CACTACT TTTCTGTGCAGTTAATACTGC-3′ (S1457A); 5′-GAATAGAAACTACCCAGCTCAAGAGG A-3′ and 5′-GAGCTCCTCTTGAGCTGGGT AGTTTCTATTC-3′(S1524A)]. All cells were collected for luminescence analysis 48 hours post-transfection, and all transfections were performed using the Attractene reagent according to the manufacturer’s instructions (Qiagen) unless noted otherwise.

### Luciferase and viability assays

Forty-eight hours post-transfection or drug treatment, luciferase activity of the Firefly luciferase was measured with Dual-Glo (for assays using Renilla luciferase) or Bright-Glo Luciferase Assay (Promega, Madison, WI, USA). Alternatively, CellTiter-Glo (Promega) was used to measure viability following the manufacturer’s recommendations. Luminescence was read from a 96-well plate on an EG&G Berthold luminometer (Berthold Technologies, Oak Ridge, TN, USA).

### GST pull-down and immunoprecipitation

GST tagged proteins were purified as described previously [59]. Constructs were washed three times with PBS + 1% Triton X-100, pelleted and resuspended in PBS + 1% Triton X-100. Bead volume was normalized between samples by the addition of extra bead slurry prepared in the same manner for each condition. Whole cell protein extract from TZM-bl cells that were transfected with Flag-Tat_101_ for 48 hours was brought up to a final volume of 500 μl with lysis buffer and 1 μg of GST-BRCA1 constructs (1–500, 504–802, 697–1276, 1021–1552, 1501–1861) were rotated at 4°C overnight. GST-alone and GST-Tat beads were washed once with TNE_150_ + 0.1% NP-40 and twice with TNE_50_ + 0.1% NP-40. GST-BRCA1 beads were washed once with TNE_300_ + 0.1% NP-40, once with TNE_150_ + 0.1% NP-40, and once with TNE_50_ + 0.1% NP-40. For IP, 1 mg of whole cell protein was brought up to a final volume of 500 μl with TNE_50_ + 0.1% NP-40 and pre-cleared for 15 min with 50 μl of 30% A/G agarose bead slurry (CalBioChem, La Jolla, CA). Supernatants were transferred to a new tube with 10 μg of BRCA1 or normal rabbit IgG antibodies (Santa Cruz), and the solution was rotated overnight at 4°C. The next day complexes were precipitated with A/G beads for 90 min. Beads were washed once with TNE_150_ + 0.1% NP-40 and twice with TNE_50_ + 0.1% NP-40. Cells were collected directly in lysis buffer and boiled for 10 min. The GST-construct plasmids pDC78 GST-BRCA1 (1–500), pDC80 GST-BRCA1 (1021–1552), pDC81 GST-BRCA1 (1501–1861), pDC99 GST-BRCA1 (504–802), pDC208 GST-BRCA1 (697–1276) were originally a kind gift from Dr. Tanya Paull at the University of Texas/ICMB [58].

### Reporter virus generation and infections

*pHCMV-G* [74], which expresses the vesicular stomatitis virus glycoprotein, and *pCMVΔR8.2* [75] have been previously described. *pNL-RRE-SA-Luc* was generated from *pNL-Luc-RRE-SA* [76] and *pNL-RRE-SA* [77] by inserting the luciferase gene (Firefly) within the XhoI cloning site of *pNL-RRE-SA*. Pseudotyped virions were prepared by co-transfecting 4×10^6^ HEK-293 T cells with 7.5 μg of packaging construct *pCMVΔR8.2*, 10 μg of reporter vector plasmid *pNL-RRE-SA-Luc*, and 2.5 μg of the envelope plasmid *pHCMV-G* using Lipofectamine 2000 (Invitrogen, Life Technologies) as recommended by the manufacturer. Viral particles were harvested 2 days post-transfection, filtered through a 0.45 μm nitrocellulose membrane, and stored at −80°C. Levels of p24 in the viral supernatant were measured by ELISA using an in-house ELISA kit. Twenty-four hours before infection, UWB1.289 and UWB1.289 + BRCA1 cells were seeded in a 96-well plate and co-transfected with 0.25 μg of *pRL-CMV-luciferase* reporter (Renilla), and pcDNA or pcTat_101_. The next day, the cells were infected in 150 μL of medium containing virus (p24 = 2,000 pg). Twenty-four hours post-infection the cells were processed for luciferase readings using the Dual-Glo system (Promega).

HIV-1 NL4-3 was generated by transfection of plasmid pNL4-3 into HEK293T cells using lipofectamine 2000 (Invitrogen) as described previously [77]. For infection, CEM T-cells were incubated with the virus (p24 = 5,000 pg/ml) for 4 hours and then washed twice with medium to remove unbound viral particles. Infected cells were resuspended in fresh RPMI supplemented with 10% heat-inactivated FBS and incubated for 72 hours prior to ChIP assays.

### Chromatin immunoprecipitation assays

Cells were crosslinked with 1% paraformaldehyde for 10 min and crosslinking was stopped by the addition of 125 mM glycine. Chromatin fragments were prepared from 5x10^6^ cells per sample. Cells were lysed using SDS lysis buffer (1% SDS, 10 mM EDTA, 50 mM Tris–HCl pH 8.0, one tablet complete protease inhibitor cocktail per 50 ml) on ice for 10 min. Cells were sonicated on ice for 6 bursts of 10 seconds to obtain an average DNA length of 500 to 1000 bp (Misonix XL 2000, Misonix, NY, USA). Samples were processed as previously described [78]. Quantitative PCR was performed using SYBR Green PCR Master Mix (#4309155, Applied Biosystems, Foster City, CA) with 5 μl of immunoprecipitated material, 0.2 μM of primer [HIV-1 LTR (−69 − +175) *Forward* 5′-CTGGGCGGGACTGGGGAG-3′ and *Reverse* 5′-TCACACAACAGACGGGCACAC-3′]. The antibodies used for immunoprecipitation were as follows: total RNAP II CTD (ab817, Abcam), pS10-H3 (Novex, Life Technologies), BRCA1 (sc-642, Santa Cruz), p-BRCA1 S1423 (sc-101647, Santa, Cruz), Sp1 (5931, Cell Signaling), (IgG (sc-2027, Santa Cruz) or V5 (AbD Serotec, Oxford, UK).

### qRT-PCR

RNA analysis of BRCA1 and ATM transcripts was performed after selective depletion with the respective siRNA. Total RNA was isolated from cell pellets using the RNeasy Kit (Qiagen) according to the manufacturer’s protocol. A total of 300 ng of RNA was used to generate cDNA with the High-Capacity RNA-to-cDNA Kit (#4387406, Invitrogen, Life Technologies) following manufacturer’s recommendations. Quantitative PCR was performed with SYBR Green PCR Master Mix Applied Biosystems). Fold changes were calculated relative to Actin using the ΔΔCt method. Primers used are described: BRCA1 *Forward* 5′-GGCTATCCTCTCAGAGTGACA TTT-3′ and *Reverse* 5′-GCTTTATCAGGTTAT GTTGCATGGT-3′ [79], ATM *Forward* 5′- CAGGGTAGTTTAGTTGAGGTTGACAG-3′ and *Reverse* 5′- CTATACTGGTGGTCAGTG CCAAAGT-3′ [80].
